# METTL14 protects sheep small intestinal epithelial cells against *Escherichia coli* F17 infection by influencing the PI3K–Akt signaling pathway

**DOI:** 10.1186/s13567-025-01627-4

**Published:** 2025-12-04

**Authors:** Qi Li, Weihao Chen, Yanjun Duan, Huiguo Yang, Kai Quan, Xiang Chen, Xiaoyang Lv, Wei Sun

**Affiliations:** 1 College of Veterinary Medicine, Yangzhou University, Yangzhou, 225009 China; 2 College of Animal Science and Technology, Yangzhou University, Yangzhou, 225009 China; 3 Institute of Animal Husbandry, Xinjiang Academy of Animal Sciences, Urumqi, 830013 China; 4 College of Animal Science and Technology, Henan University of Animal Husbandry and Economics, Zhengzhou, 450046 China; 5 Guizhou Provincial Key Laboratory of Animal Genetics, Breeding and Reproduction, Guizhou University, Guiyang, 550025 China; 6 Joint International Research Laboratory of Agriculture and Agri-Product Safety of Ministry of Education, Yangzhou University, Yangzhou, 225009 China; 7 International Joint Research Laboratory in Universities of Jiangsu Province of China for Domestic Animal Germplasm Resources and Genetic Improvement, Yangzhou, 225009 China

**Keywords:** *Escherichia coli* F17, METTL14, sheep diarrhea

## Abstract

*Escherichia coli* F17 is emerging as one of the main causes of bacterial diarrhea in sheep. After lambs are infected, the bacteria colonize the small intestinal epithelial cells (IECs) through adhesion of F17 fimbriae. This colonization triggers the release of enterotoxins, disrupting the intestinal mucosa and resulting in symptoms such as diarrhea. These pathological effects contribute to considerable global economic losses and continuously impact the economic development of the sheep farming industry. Meanwhile, m^6^A methylation, the most prevalent form of messenger RNA (mRNA) modification, participates in fundamental pathophysiological and metabolic processes of RNAs. Methyltransferase-like 14 (METTL14), a key component of the m^6^A methyltransferase complex, has been identified as playing an important role in cell migration and invasion. In addition, METTL14 has been demonstrated to contribute to the antimicrobial mechanisms of cells. However, its potential role in IECs in *E. coli* F17-infected sheep remains unclear. In this study, we demonstrate that METTL14 regulates the susceptibility of sheep small intestinal epithelial cells to *E. coli* F17. Specifically, we observed that METTL14 expression was significantly increased in sheep IECs following *E. coli* F17 infection. Using bacterial adhesion assays (bacterial counting, Gram staining, and fimbrial gene expression analysis), together with vimentin protein detection, western blotting, and scratch tests, we demonstrate that METTL14 overexpression or knockdown affects the susceptibility of sheep IECs to *E. coli* F17. Furthermore, RNA-seq analysis after METTL14 knockdown, combined with pathway inhibition experiments, revealed that the PI3K–Akt signaling pathway is involved in the anti-adhesion response. Blocking this pathway confirmed that METTL14 modulates the susceptibility of IECs to *E. coli* F17 via PI3K–Akt signaling. These findings provide new insights into the pathogenic mechanism of *E. coli* F17 and establish an experimental basis for enhancing sheep resistance to *E. coli*-related diseases.

## Introduction

Diarrhea remains a major problem in the global livestock industry, and enterotoxin-producing *Escherichia coli* F17 (*E. coli* F17) is emerging as one of the main causes of bacterial diarrhea in sheep, often occurring alongside *Salmonella* spp. [1, 2]. When lambs are infected, they frequently die from severe watery diarrhea and rapid dehydration, with morbidity and mortality rates remaining high [3]. Owing to the continuing emergence of multidrug-resistant strains, the global economic impact is large and the development of the sheep farming industry suffers significant losses [4, 5]. The pathogenic process of *E. coli* F17 is driven primarily by adhesins and enterotoxins [6]. F17-like fimbriae typically colonize the epithelial cells of sheep IECs, and the colonized bacteria produce toxins that lead to electrolyte loss, eventually causing watery diarrhea. This process also involves the release of enterotoxins, which triggers a series of symptoms such as diarrhea in lambs [7, 8]. Clearly, adhesion of *E. coli* to host cells is a key factor in this pathogenesis [9]. At the same time, RNA modification serves as an important epigenetic mechanism in post-transcriptional regulation and is widely observed in animals, plants, and microorganisms [10]. Current studies show that RNA modification influences RNA function, protein abundance, and diverse biological processes—such as cell differentiation, development, and stress responses—by regulating RNA splicing, stability, and translation efficiency [11]. Among these modifications, m^6^A methylation emerges as the most common form of mRNA modification, being present in nearly all eukaryotes as well as some bacteria, viruses, yeasts, and plants [12]. m^6^A methylation occurs broadly in mRNAs, miRNAs, and long noncoding RNAs and plays a role in fundamental pathophysiological and metabolic processes, including RNA splicing, nuclear export, translation, decay, folding, and RNA–protein interactions [13, 14]. Moreover, m^6^A methylation functions as a dynamic and reversible co-transcriptional process in which m^6^A modifications are added by “writers” (N^6^-methyladenosine methyltransferases) [15, 16], removed by “erasers” (demethylases) [17], and recognized by “readers” (m^6^A-binding proteins) [18]. Among these, m^6^A modifications are primarily mediated by m^6^A methyltransferases (writers), including methyltransferase-like 14 (METTL14), methyltransferase-like 3 (METTL3), and Wilms’ tumor 1-associated protein (WTAP) [19]. METTL3 is recognized as the only catalytically active methyltransferase, but it must bind to METTL14 to function effectively [20]. Although METTL14 lacks true catalytic activity, it still serves as a critical component for METTL3 activity, enhancing its methyltransferase function by recognizing RNA substrates and guiding methylation localization [21]. As a key component of the m^6^A methyltransferase complex, the METTL14 gene is frequently identified as playing an important role in cell migration and invasion [22]. For example, METTL14 has been shown to promote cell proliferation and migration by directly targeting PERP in an m^6^A-dependent manner [23]. Another study found that METTL14 overexpression inhibits papillary thyroid carcinoma (PTC) cell proliferation and migration/invasion by suppressing OIP5-AS1 expression and regulating the epidermal growth factor receptor (EGFR) and mitogen-activated protein kinase kinase (MEK)/extracellular signal-regulated kinase (ERK) pathways [24]. Additionally, a small number of studies have suggest that METTL14 also plays a role in resistance to bacterial infections, although research in this area remains limited [25].

## Materials and methods

### Bacterial strains and cells

The *E. coli* F17 strain (DN1502) used in this study was provided by Prof. Dongfang Shi from Northeast Agricultural University (Harbin, China). Sheep small intestinal epithelial progenitor cells were isolated, purified, and preserved in our laboratory.

### Cell culture and *E. coli* F17 stimulates sheep IECs

Sheep IECs were cultured in Dulbecco’s modified Eagle’s medium (DMEM)/F12 medium supplemented with 10% fetal bovine serum and 1% penicillin–streptomycin in a 5% CO₂ incubator at 37 °C. Cell growth was monitored using an inverted fluorescence microscope. *E. coli* F17 was inoculated onto lysogeny broth (LB) agar plates and incubated at 37 °C for 16 h. Single colonies were then picked with an inoculation loop and transferred into LB liquid medium, followed by incubation at 180 rpm for 16–18 h at 37 °C in a shaker. The optimal stimulation conditions for *E. coli* F17 had been previously established in this laboratory [26]. The procedure was as follows: When the cells reached 80–90% confluency, *E. coli* F17 was pre-inoculated into LB liquid medium at a 1:1000 ratio and incubated at 220 rpm for 12 h. The culture was centrifuged at 4000 rpm for 5 min, and the bacterial pellet was collected. The pellet was then resuspended in phosphate-buffered saline (PBS) buffer, mixed, and centrifuged again. This washing step was repeated three times. Finally, the bacterial pellet was diluted to 1.0 × 10⁹ colony-forming units (CFU)/mL using DMEM/F12 culture medium. A volume of 2 mL of the diluted bacterial solution was added to each cell culture well (with three replicates per group). A blank control group was also set up, where only the cell culture medium was added. After 4 h of stimulation, the cells were collected for further analysis.

### RT-qPCR

After stimulation with *E. coli* F17, the cells were collected, and RNA was extracted using the TRIzol method. The quality and integrity of the RNA were assessed using a Nanodrop 2000 spectrophotometer (Thermo Scientific, USA). Total RNA (1 μg) from each sample was reverse-transcribed into first-strand complementary DNA using the FastKing gDNA Dispelling RT SuperMix Kit (Tiangen, Nanjing, China). Primers were designed using Beacon Designer, and the mRNA expression level of METTL14 in sheep IECs was quantified using the ChamQ SYBR qPCR Master Mix kit (Vazyme, Nanjing, China), with β-actin [27] serving as the internal reference gene (Table 1).

**Table 1:** **Primers used for RT-qPCR**

Gene name	Sequences (5′ → 3′)	Product length (bp)	Annealing temperature (°C)	Accession no.
*METTL14*	F: GGCTTCCTATGATACCTCTG R: TCCTTATATTCTTCCATCTTGTCT	90	60	XM 004009592.6
*β-actin*	F: CAGTCGGTTGGATCGAGCAT R: AGAAGGAGGGTGGCTTTTGG	151	60	NM 001009784.3
*ITGA4*	F: CTACAACGTGGACACGGAGA R: CGACTACGAGCCATCGGTT	110	60	XR 011570628.1
*ITGA11*	F: TGCAGTACGGAGAAGACGTG R: CTGGAAAGCCTCTGAGCGAG	155	60	XM 069597661.1
*CD19*	F: GTGCCAATCATGAGGAAGATGC R: ATGGTCTTTCGCCAACCTGA	174	60	XR 009598459.1
*FGF1*	F: AGAGCCAAAGCAGAGCCTTGA R: GAGGAGCTTGGGCTTCTTGTA	136	60	XM_069592295.1

### RNA oligonucleotides and plasmid construction

#### Construction of METTL4 overexpression plasmid

Primers with pcDNA3.1 homology arms were designed using Primer5 on the basis of the coding sequence (CDS) of METTL14 from the National Center for Biotechnology Information (NCBI) database (Table 2). The pcDNA3.1 plasmid was digested using restriction endonucleases QuickCut BamHI and NotI (Takara). The target fragment was amplified using PrimeSTAR Max DNA polymerase (Takara), and the amplified product was purified with the Gel Recovery Kit (Tiangen, Nanjing, China). The target fragment was ligated into the pcDNA3.1 vector using a homologous recombination kit (Vazyme, Nanjing, China) and then transformed into DH5α competent cells. Colonies were picked the next day for polymerase chain reaction (PCR) identification. Plasmid DNA was extracted from the positive clones and sent to Qingke Biotechnology Co., Ltd. (Nanjing, China) for sequencing. After sequencing, the results were compared with the target gene sequence.

**Table 2:** **Primers used for recombinant plasmid construction**

Gene name	Sequences (5′ → 3′)	Product length (bp)	Annealing temperature (°C)
*METTL14*	F: AAGCTTGGTACCGAGCTCGGATCCATGCGCGTGCGCCGCGTT R: ACGGGCCCTCTAGACTCGAGCGGCCGCCTATCGAGGTGGAAAGCCACC	1566	60

### Cell transfection

Small interfering RNA (siRNA) targeting METTL14, along with a negative control (NC), was designed and synthesized by GenePharma (Suzhou, China) (Table 3).

**Table 3:** **Oligonucleotide sequences for siRNA**

Fragment name	Sequences (5′ → 3′)
METTL14-sheep-298	GCAUUGGUGCUGUGUUAAATT UUUAACACAGCACCAAUGCTT
METTL14-sheep-838	GCUGGACUUGGGAUGAUAUTT AUAUCAUCCCAAGUCCAGCTT
METTL14-sheep-1485	GGGCGAGAGAGAAAUCGAUTT AUCGAUUUCUCUCUCGCCCTT
METTL14-sheep-3518	GCUGCACCAGCUAAUACUUTT AAGUAUUAGCUGGUGCAGCTT
NC	UUCUCCGAACGUGUCACGUTT ACGUGACACGUUCGGAGAATT

### Changes in vimentin protein mRNA levels after METTL14 overexpression and knockdown

The ChamQ SYBR qPCR Master Mix kit (Vazyme, Nanjing, China) was used to assess the efficiency of overexpression and knockdown transfection. Additionally, it was employed to investigate the effect of METTL14 on the mRNA expression levels of vimentin in sheep IECs.

### *E. coli* F17 adherence assay

The methods for cell culture, transfection, and bacterial stimulation were described in the section on cell transfection. The ability of *E. coli* F17 to adhere to sheep IECs was evaluated through bacterial counting, Gram staining, and relative quantification of the expression of *E. coli* fimbrial genes [8].

### Western blot assay

Sheep IECs were cultured in six-well plates and lysed with radioimmunoprecipitation assay (RIPA) lysis buffer (Beyotime, Shanghai, China) to extract proteins. Protein concentration was determined using a bicinchoninic acid (BCA) kit (Vazyme, Nanjing, China), and the proteins were denatured according to their concentration. After denaturation, the protein samples underwent 10% polyacrylamide gel electrophoresis to separate the target proteins, which were subsequently transferred to polyvinylidene fluoride (PVDF) membranes (Solarbio, Beijing, China). The PVDF membranes were incubated with primary antibodies, including rabbit polyclonal anti-METTL14 (1:2000), rabbit polyclonal anti-vimentin (1:5000), and mouse monoclonal anti-glyceraldehyde 3-phosphate dehydrogenase (GAPDH) (1:10 000). The membranes were then incubated with goat anti-rabbit immunoglobulin G (IgG) and goat anti-mouse IgG secondary antibodies. Detection was carried out using an enhanced chemiluminescence (ECL) Western Blot kit (BioSharp, Hefei, China).

### Scratch assay

Sheep IECs were seeded into 12-well plates and transfected when the cells reached 60% confluence. Prior to transfection, a scratch assay was performed. A horizontal line was drawn on the back of each well of the six-well plate using a marker, and scratches were then made perpendicular to the line in each well using a ruler. After scratching, transfection was carried out, and the migration ability of the cells was analyzed on the basis of their healing speed from 0 to 12 h.

### RNA-seq analysis

The methods for cell culture, transfection, and bacterial stimulation were described in the section on cell transfection. Sheep IECs were cultured in six-well plates to collect RNA-seq samples and divided into two groups: the *E. coli* F17 stimulation group and the NC group, with two replicates per group. After 36 h of siRNA transfection, the cells were collected to obtain four RNA-seq samples for total RNA extraction. Total RNA was extracted using the TRIzol kit (Invitrogen, Carlsbad, CA, USA) and subsequently sent to Shanghai Personalbio for transcriptome sequencing.

### Bacterial adhesion and cell migration assays performed after blocking the signaling pathways using pathway inhibitors

Cell experiments were performed using the PI3K–AKT pathway inhibitor (MedChemexpress, America) according to the manufacturer’s instructions, and the experiments were divided into four groups: the PI3K–AKT pathway inhibitor group, the PI3K–Akt pathway inhibitor + METTL14 overexpression plasmid co-transfection group, the METTL14 overexpression plasmid transfection group, and the pcDNA3.1 empty vector group. The specific steps were as follows: cells were cultured in six-well plates, and when the cell density reached 70%, the cytostatic inhibitor was added, with a final concentration of 0.04 μM per well. Colony counting, Gram staining, and cell migration experiments were then performed.

## Results

### The effect of *E. coli* F17 stimulation on METTL14 mRNA expression levels in sheep IECs

**Figure 1:**
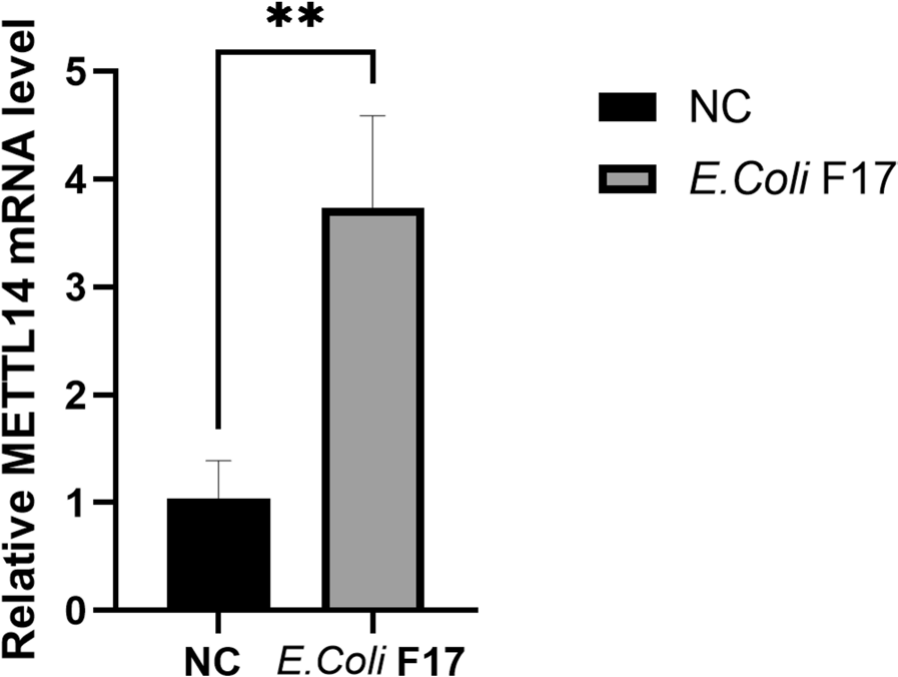
**mRNA expression of METTL14 in sheep IECs after *E. coli*** **F17 stimulation.**Relative mRNA levels of METTL14 in sheep intestinal epithelial cells (IECs) after 4 h of stimulation with *E. coli* F17. *P* < 0.01.

The expression levels of METTL14 in IECs are shown in Figure 1. Compared with the control group, the *METTL14* gene expression in the cells increased significantly after *E. coli* F17 stimulation (*p* < 0.01).

### Efficiency detection of overexpression and interference of METTL14

**Figure 2:**
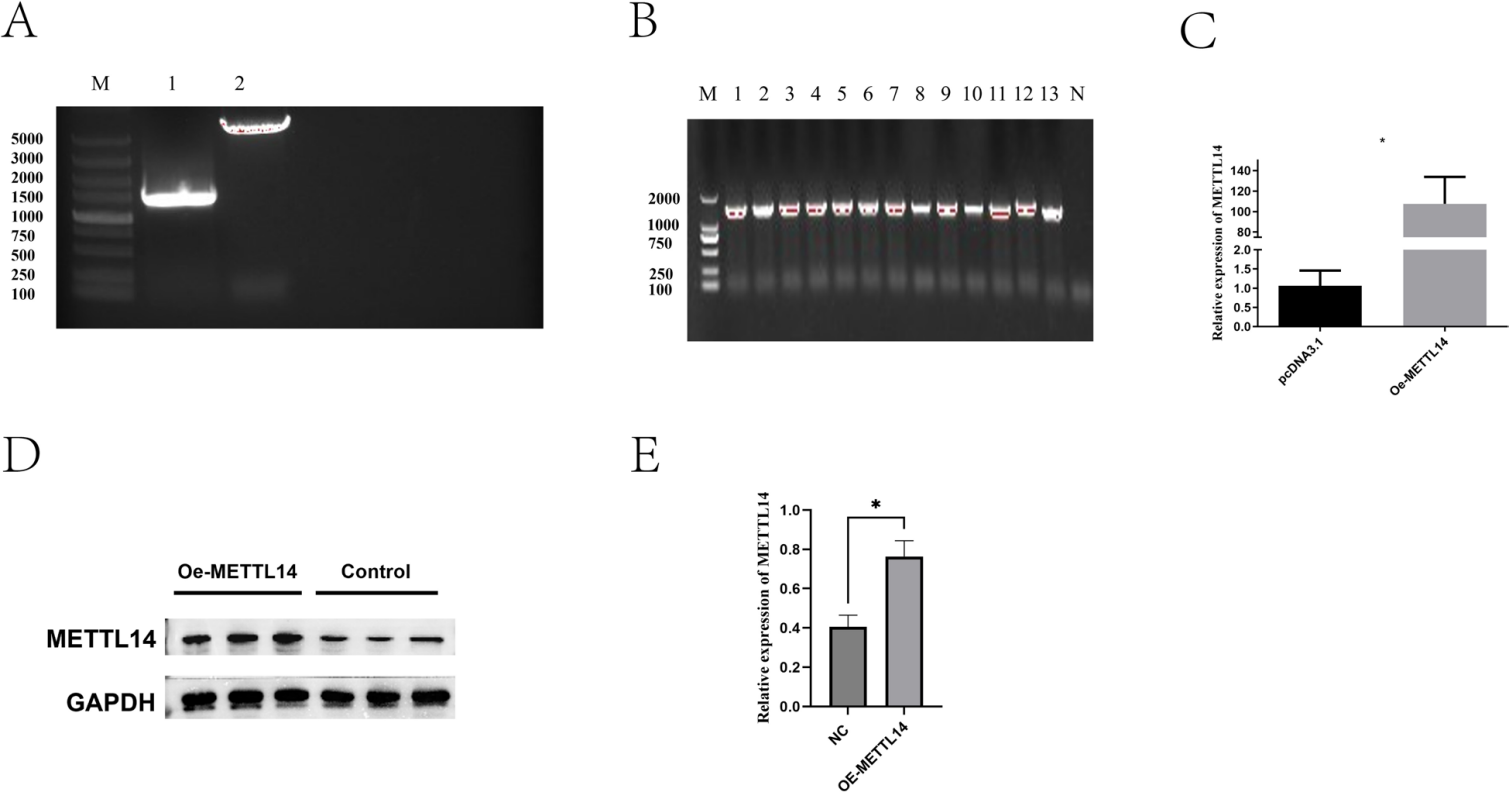
**Construction and validation of METTL14 overexpression vector.** **A** Amplified target fragment of METTL14; **B** Double enzyme digestion of the pcDNA3.1 vector; **C** PCR identification of recombinant plasmid colonies; **D** Detection of METTL14 mRNA levels after transfection; **E**, **F** Western blot analysis of METTL14 protein levels after transfection. *P* < 0.05.

METTL14 was cloned into the pcDNA3.1 vector using a homologous recombination kit. The construct was verified by colony PCR and confirmed by Sanger sequencing, demonstrating the successful construction of the METTL14 overexpression vector (Figures 2A, B). The mRNA and protein expression levels of METTL14 in sheep IECs were analyzed using RT-qPCR and western blot, respectively. The results showed that transfection with the METTL14 overexpression plasmid significantly increased METTL14 expression at the mRNA level (Figure 2C) (*p* < 0.5). Additionally, western blot analysis revealed that the protein levels in the METTL14 overexpression group were significantly higher than those in the empty vector group (Figures 2D, E) (*p* < 0.5). These results indicate that the METTL14 overexpression plasmid and siRNA were successfully transfected and could be used for subsequent analyses.

**Figure 3:**
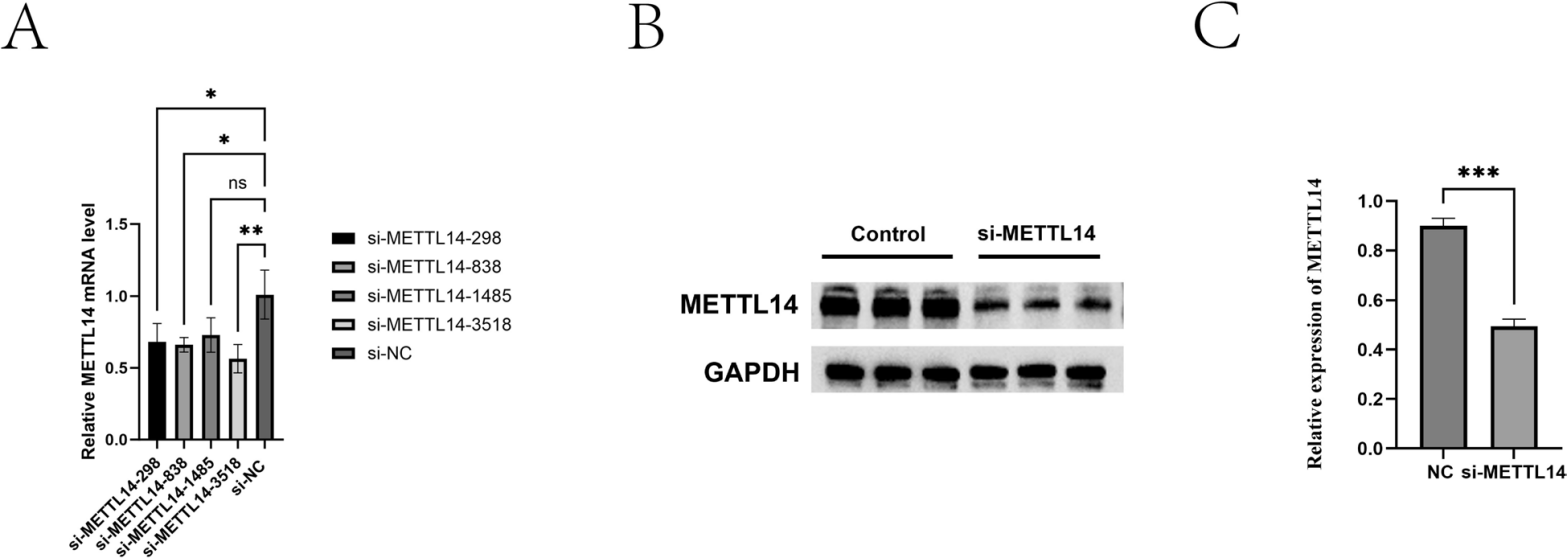
**Efficiency of METTL14 knockdown in sheep IECs.** **A** mRNA expression levels of METTL14 after siRNA transfection; **B**, **C** Western blot analysis of METTL14 protein expression after knockdown. *P* < 0.01, *P* < 0.001.

The interference efficiency was determined by RT-qPCR, identifying si-METTL14-838 as the optimal interference sequence (Figure 3A). Western blot analysis was performed to detect the mRNA and protein expression levels of METTL14 in sheep IECs. The results showed that, after transfection with si-METTL14, METTL14 expression was significantly reduced at both the mRNA and protein levels (Figures 3B, C), which was the opposite of the overexpression results.

### METTL14 influences the *E. coli* F17 susceptibility of sheep IECs

**Figure 4:**
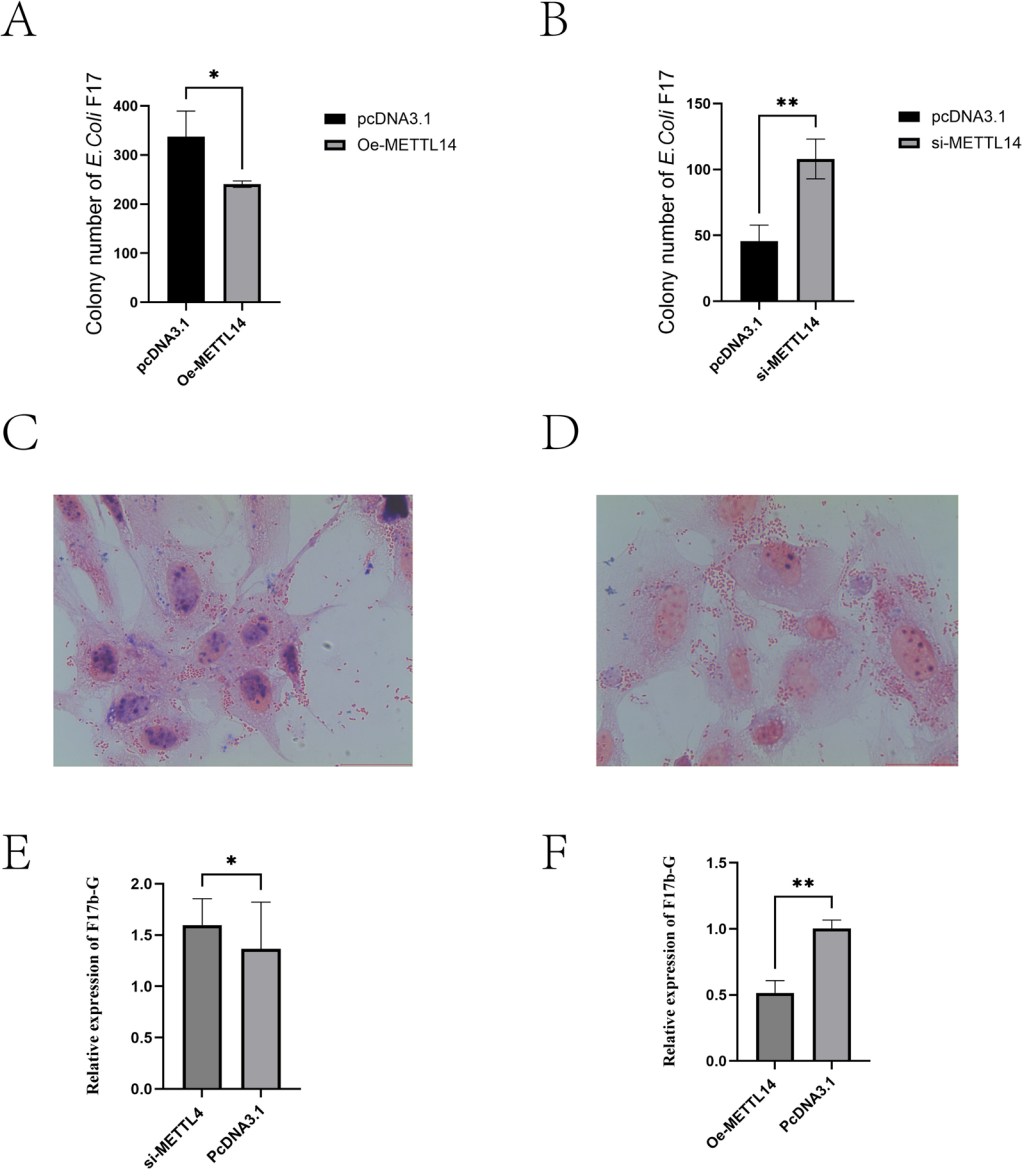
**Effect of METTL14 on susceptibility of sheep IECs to** ***E. coli*** **F17**. **A** Bacterial counts after METTL14 overexpression; **B** Bacterial counts after METTL14 knockdown; **C** Gram staining after METTL14 overexpression; **D** Gram staining after METTL14 knockdown; **E** Relative fimbrial gene expression after METTL14 knockdown; **F** Relative fimbrial gene expression after METTL14 overexpression. *P* < 0.05, *P* < 0.01.

*E. coli* F17 infection assays were performed in sheep IECs transfected with Oe-METTL14, pcDNA3.1, si-METTL14, and si-NC. The effects of METTL14 on *E. coli* F17 infection were verified by *E. coli* F17 colony counting, Gram staining, and fimbrial gene RT-qPCR, respectively, to assess the role of METTL14 in sheep IECs in *E. coli* F17 infection. The results of colony counting (Figures 4A, B) showed that *E. coli* F17 adhesion to IECs in the Oe-METTL14 transfection group was significantly lower than in the pcDNA3.1-NC group (*P* < 0.05). Conversely, *E. coli* F17 adhesion to IECs in the si-METTL14 transfection group was significantly higher than in the si-NC group (*P* < 0.01).

The Gram staining experiment showed that the number of *E. coli* F17 bacteria adhering to IECs in the Oe-METTL14 transfection group was significantly lower than in the si-METTL14 transfection group (Figures 4C, D).

RT-qPCR targeting the fimbrial gene F17b-G was performed, and the results (Figure 4E) indicated that F17b-G fimbrial gene expression was significantly higher in the si-METTL14 transfection group compared with the si-NC group (*P* < 0.05). In contrast (Figure 4F), F17b-G fimbrial gene expression was lower in the Oe-METTL14 transfection group than in the NC group (*P* < 0.01).

### The effect of METTL14 on the mRNA and protein expression levels of vimentin

**Figure 5:**
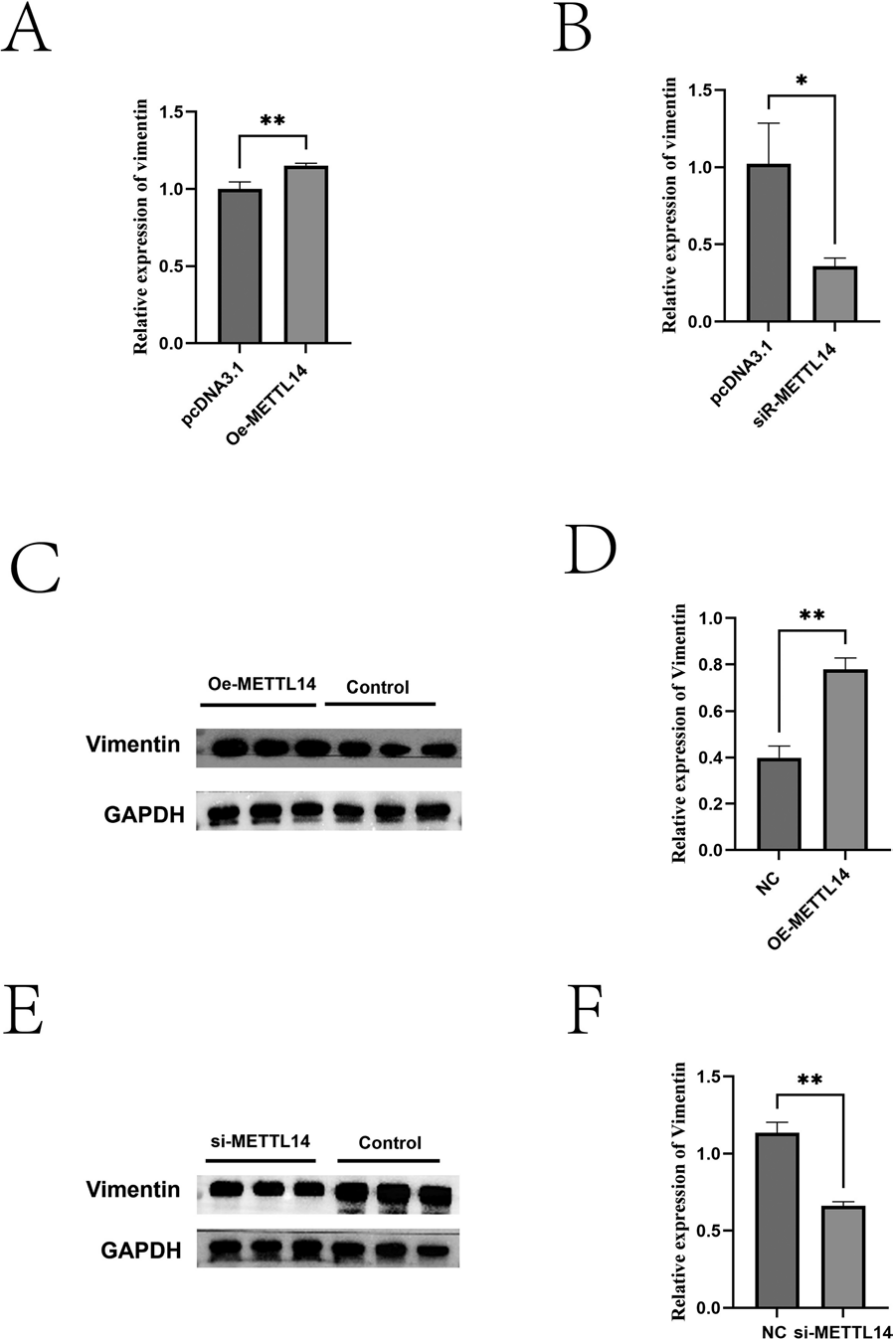
**Effect of METTL14 on vimentin expression in sheep IECs.** **A** mRNA expression levels of vimentin after METTL14 overexpression; **B** mRNA expression levels of vimentin after METTL14 knockdown; **C**, **D** Western blot analysis of vimentin protein after METTL14 overexpression; **E**, **F** Western blot analysis of vimentin protein after METTL14 knockdown. *P* < 0.05, *P* < 0.01.

RT-qPCR and western blot analyses showed that the vimentin expression in the Oe-METTL14 group was significantly higher than that in the control group at both mRNA and protein levels (Figures 5A, C, D) (*P* < 0.01). In contrast, the vimentin expression in the METTL14 interference group was significantly lower than that in the control group at both mRNA and protein levels (Figures 5B, E, F) (*P* < 0.01).

### METTL14 promotes the migration of IECs

**Figure 6:**
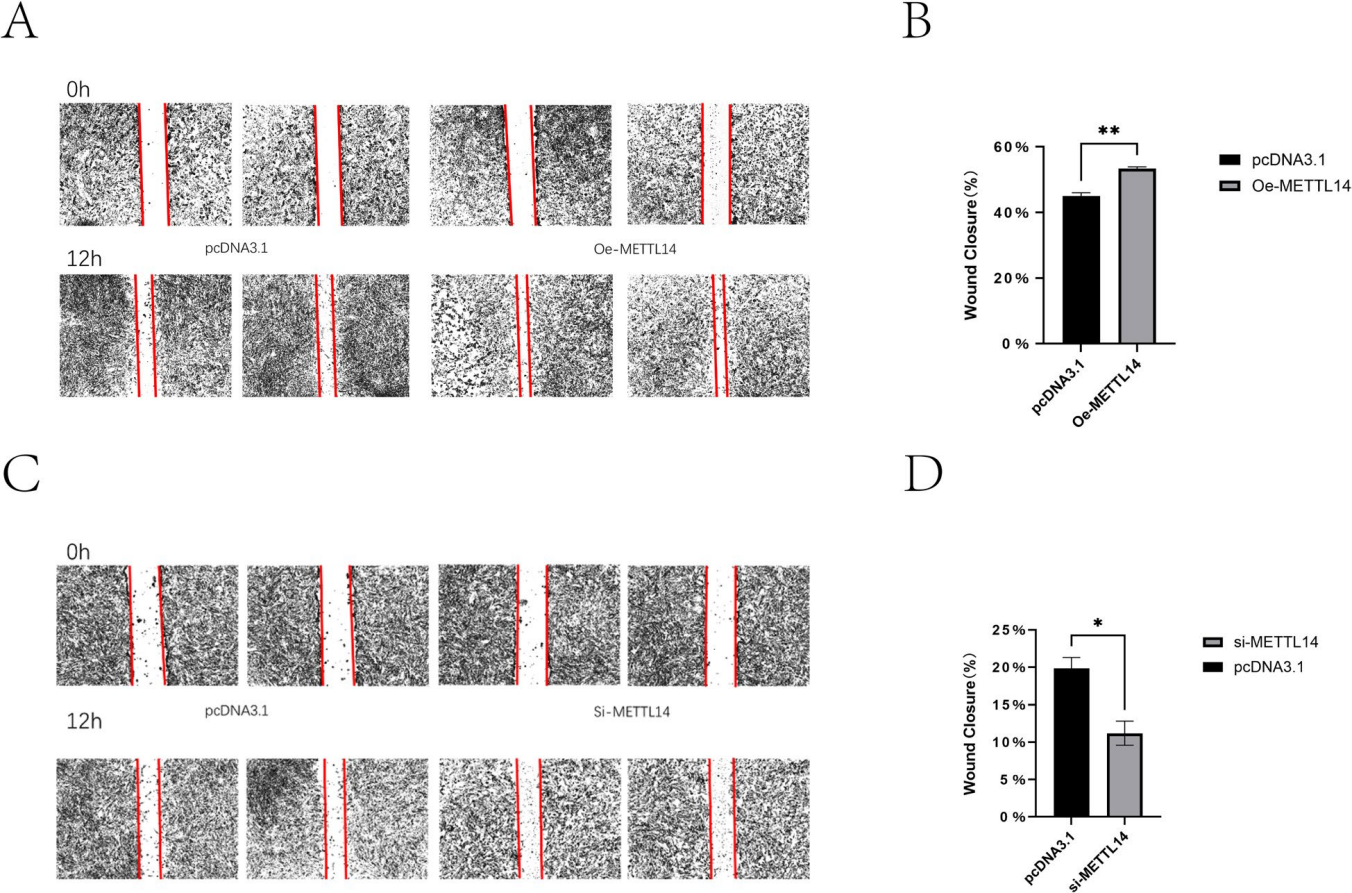
**Effect of METTL14 on IEC migration.** **A**, **B** Scratch assay results after METTL14 overexpression; **C**, **D** Scratch assay results after METTL14 knockdown. *P* < 0.05, *P* < 0.01.

In this study, the effect of METTL14 on the migration of sheep IECs was examined using a cell scratch assay. The results showed that the wound healing speed of cells in the transfected Oe-METTL14 group was significantly faster than that in the pcDNA3.1 group (Figures 6A, B) (*P* < 0.05), whereas the wound healing speed of cells in the transfected si-METTL14 group was significantly slower than that in the si-NC group (Figures 6C, D) (*P* < 0.05), indicating that METTL14 promoted the migration of sheep IECs.

### METTL14 regulates the expression of downstream genes

**Figure 7:**
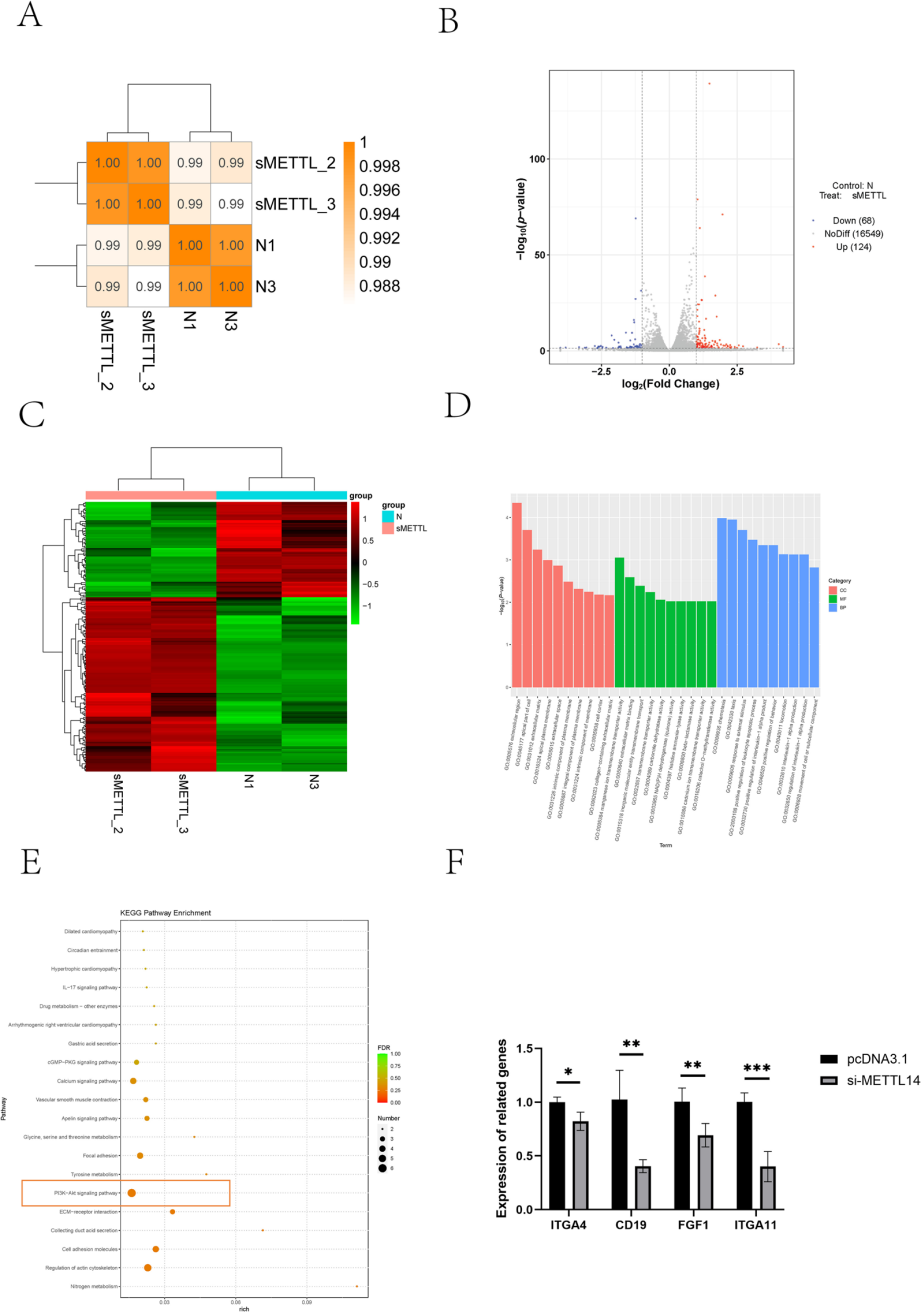
**Transcriptomic analysis after METTL14 knockdown in sheep IECs.** **A** Correlation analysis of the samples; **B** Volcano plot of differentially expressed genes (DEGs); **C** Heatmap of significantly expressed genes; **D** GO enrichment analysis of DEGs; **E** KEGG enrichment analysis of DEGs; **F** mRNA levels of PI3K–Akt pathway-related genes after METTL14 knockdown. *P* < 0.05, *P* < 0.01,* P* < 0.001.

To further investigate the molecular mechanism by which METTL14 regulates the resistance of IECs to *E. coli* F17, RNA-seq was performed after METTL14 interference. Following sequencing quality control, Q20, Q30, GC content, and the ratio of clean reads were evaluated for each sample. Correlation analysis revealed a high degree of similarity among replicate samples within the same group, indicating that the RNA-seq data were of high quality and suitable for further analysis (Figure 7A).

To identify differentially expressed genes (DEGs) between the two groups (si-METTL14 versus si-NC), differential expression was analyzed using the following criteria: fold change ≥ 2 and false discovery rate (FDR) < 0.05. A total of 192 DEGs were identified, including 124 upregulated and 68 downregulated genes. A volcano plot was used to visualize differences in gene expression levels and their statistical significance (Figure 7B).

Hierarchical clustering was performed on the 124 upregulated and 68 downregulated genes. The analysis showed that samples from the same group clustered closely, further confirming the accuracy and reliability of the sequencing data (Figures 7C, D).

On the basis of the KEGG enrichment analysis results of DEGs obtained from RNA-seq, significant enrichment was found in the PI3K–Akt signaling pathway (Figure 7E), with four differentially expressed genes (DEGs) identified as being enriched in this pathway (Figure 7F). This finding prompted further investigation into the role of METTL14 in the PI3K/AKT signaling pathway and its impact on cellular resistance to *E. coli* F17.

### METTL14 enhances the PI3K–Akt pathway in sheep IECs

To investigate the effect of METTL14 on cellular resistance to *E. coli* F17 infection via the PI3K–Akt pathway, cell experiments were conducted using pathway inhibitors. Four groups were established: the pcDNA3.1 group, the PI3K–Akt pathway inhibitor group, the PI3K–AKT inhibitor + METTL14 group, and the Oe-METTL14 group. Validation was performed using methods such as bacterial counting, Gram staining, relative quantification of fimbrial gene expression, and scratch assays.

**Figure 8:**
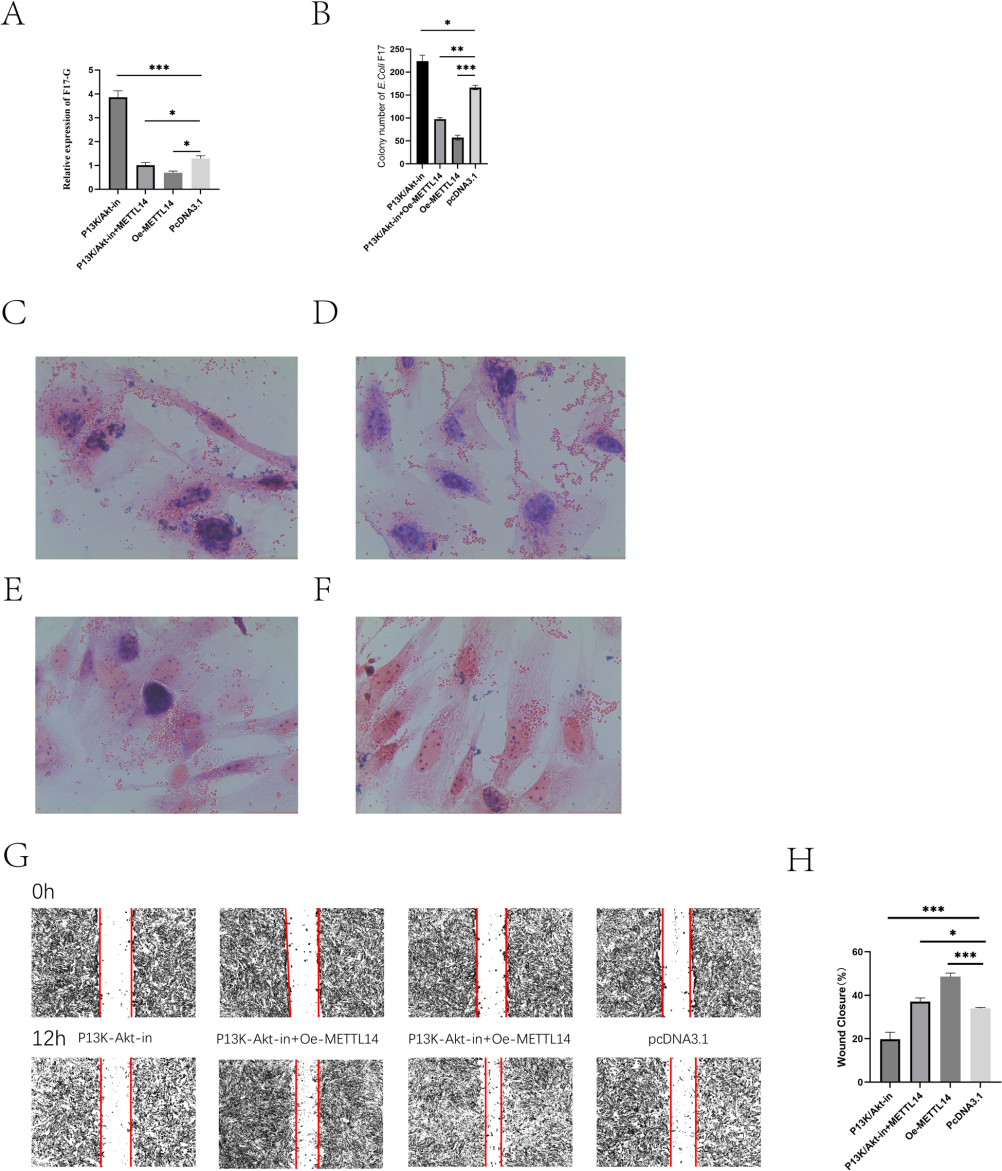
**METTL14 regulates the susceptibility of sheep IECs to** ***E. coli* F17 via the PI3K–Akt pathway.** **A** qPCR analysis of fimbrial gene expression after PI3K–Akt pathway inhibition; **B** Bacterial counts after PI3K–Akt pathway inhibition; **C–F** Gram staining results of pcDNA3.1, PI3K–Akt inhibitor, PI3K–Akt inhibitor + METTL14, and Oe-METTL14 groups; **G**, **H** Cell migration after PI3K–Akt pathway inhibition. *P* *< 0.05,* *P < 0.01,* *P* *< 0.001.*

The results indicated that, after PI3K–Akt pathway inhibition, RT-qPCR analysis showed that the highest level of fimbrial protein was observed in the PI3K–Akt pathway inhibitor group, followed by the METTL14 + PI3K–Akt pathway inhibitor group, the empty vector group, and the METTL14 overexpression group (Figure 8A).

Colony counting results showed that inhibition of the PI3K–Akt signaling pathway significantly increased the adhesion of *E. coli* F17. Co-transfection of the PI3K–Akt pathway inhibitor and the METTL14 overexpression plasmid slightly reduced bacterial adhesion, while overexpression of the METTL14 gene significantly decreased bacterial adhesion (Figure 8B). The Gram staining results were consistent with these findings (Figures 8C–F).

Scratch assay results demonstrated that inhibition of the PI3K–Akt signaling pathway significantly reduced the cell healing ability. Co-transfection of the PI3K–Akt pathway inhibitor and the METTL14 overexpression vector slightly improved the cell healing ability, while overexpression of the METTL14 gene significantly enhanced cell healing ability (Figures 8G, H).

These findings indicate that inhibition of the PI3K–Akt signaling pathway reduces the resistance of IECs to *E. coli* F17 and enhances bacterial adhesion. However, overexpression of METTL14 can restore the resistance of IECs to *E. coli* F17, reduce bacterial adhesion, and improve the cell healing ability.

## Discussion

Sheep bacterial diarrhea caused by *Escherichia coli* F17 infection is a serious problem in modern sheep farming and has been reported in many countries, including Peru, Russia, and Iran [28, 29]. Current prevention and control strategies mainly rely on antibiotics. However, owing to the excessive and irregular use of antibiotics in some farms, multidrug-resistant *E. coli* strains have emerged at an alarming rate [30–32]. Shabana et al. isolated multiple *Escherichia coli* strains containing resistance genes from diarrheic goats, with a resistance rate to cephalosporins as high as 44% [33]. Therefore, in addition to pathogen-based control, long-term prevention should focus on understanding the molecular mechanisms underlying host resistance and exploring genetic strategies for disease-resistant breeding [34–37]. RNA m^6^A modification is an important epigenetic regulatory mechanism that influences diverse physiological processes, including immunity and host–pathogen interactions. Previous work demonstrated that the methyltransferase METTL3 enhances resistance to *E. coli* F18 by activating the IKBKG–NF-κB signaling axis [38]. In the present study, we observed a significant upregulation of METTL14 expression in sheep intestinal epithelial cells (IECs) after *E. coli* F17 challenge. These findings suggest that METTL14 may act as a novel regulator of host defense against pathogenic *E. coli*. Bacterial adhesion is the first critical step for infection, and *E. coli* F17 relies on fimbrial structures to attach to specific receptors on host intestinal epithelial cells [39, 40]. Host susceptibility to infection has been shown to depend on the expression of epithelial receptors and the regulation of immune-related genes. For example, bovine lactoferrin competes with *E. coli* for binding sites, thereby reducing colonization [41]. The resistance of animals such as pigs and cattle to *Escherichia coli* depended on the expression of corresponding receptors in their intestinal epithelial cells and the ability of certain genes to regulate intestinal immunity [42, 43]. Similarly, FUT8 deficiency in pigs confers resistance to *E. coli* F18 [44]. Consistent with these studies, our findings revealed that METTL14 overexpression significantly reduced bacterial adhesion, while METTL14 knockdown promoted adhesion, supporting a protective role for this methyltransferase in host defense. Further mechanistic analysis suggested that METTL14 regulates the expression of vimentin, a cytoskeletal protein that is increasingly recognized as a multifunctional regulator in host–pathogen interactions [45–47]. Vimentin not only participates in cytoskeletal organization and cellular migration but also acts as a receptor or cofactor for bacterial and viral invasion [48]. Recent studies have shown that vimentin played a role in regulating the host’s inflammatory response, and type I interferon (IFN-I) and type III interferon (IFN-III) could also upregulate vimentin expression [49]. Furthermore, studies have demonstrated that demonstrated that METTL3 overexpression increased vimentin expression in human non-small cell lung cancer cells [50]. Here, we provide the first evidence that METTL14 similarly modulates vimentin expression in sheep IECs, which may contribute to the observed reduction in bacterial adhesion and the enhancement of epithelial repair capacity.

In addition to vimentin, our study revealed that METTL14 exerts its regulatory effects through the PI3K–Akt signaling pathway. This pathway is one of the most important intracellular cascades controlling cell survival, proliferation, migration, and stress responses [51, 52]. The innate immune response is a crucial mechanism for the host to resist the invasion of pathogenic microorganisms. During pathogen invasion, the host utilizes the innate immune system to combat the intrusion of these microorganisms. Studies have found that the expression of ST3GAL1 in porcine intestinal epithelial cells regulated the cells’ resistance to *Escherichia coli* F18 infection. ST3GAL1 enhanced innate immunity against *E. coli* infection by activating the TLR signaling pathway in porcine intestinal epithelial cells during *E. coli* F18 infection [53]. Previous work also showed that METTL14 could modulate cancer cell progression by targeting PI3K–Akt [54]. Our RNA-seq analysis confirmed that PI3K–Akt signaling was significantly enriched in METTL14-regulated cells, and functional assays demonstrated that pathway inhibition increased bacterial adhesion and impaired epithelial healing. Interestingly, METTL14 overexpression could partially rescue these defects, highlighting a key role for METTL14 in sustaining PI3K–Akt–mediated epithelial defense against *E. coli* F17.

Taken together, our findings suggest a dual mechanism by which METTL14 protects sheep IECs from *E. coli* F17 infection: first, by regulating vimentin expression to influence cellular resistance to bacterial infection; and second, by activating the PI3K–Akt pathway to promote cell survival, migration, and repair. This suggests that METTL14 may play a potential role in the resistance of sheep IECs to *E. coli*. This not only expands our understanding of m^6^A methyltransferase function in host–pathogen interactions but also identifies METTL14 as a promising molecular target for developing novel strategies to enhance disease resistance in sheep.

Nevertheless, our study has certain limitations. For instance, owing to high cell mortality upon bacterial challenge, we were unable to perform cell migration assays under infection conditions. Future work should optimize experimental approaches to further clarify the contribution of METTL14 to epithelial repair in the presence of bacterial pathogens. In addition, in vivo studies will be necessary to confirm the physiological relevance of our in vitro findings.

## Conclusions

In this study, upregulation of METTL14 significantly reduced the adhesion ability of *Escherichia coli* F17 to cells and markedly enhanced the cells’ resistance to *E. coli* F17. After knocking down METTL14 and exposing cells to *E. coli* F17, combined with RNA-seq and experimental validation, we further revealed that METTL14 can influence the PI3K–Akt signaling pathway and rescue its inhibition. This enhances the immune capacity of cells, thereby regulating the susceptibility of sheep small intestinal epithelial cells to *E. coli* F17.

## Data Availability

All data generated or analyzed during this study are included in this published article and its supplementary information files.

## Abbreviations

WB: western blotting
METTL3: methyltransferase-like 3
METTL14: methyltransferase-like 14
WTAP: Wilms’ tumor 1-associated protein
PTC: papillary thyroid carcinoma
siRNA: small interfering RNA
NC: negative control
DEGs: differentially expressed genes
FDR: false discovery rate
